# Fast Detection and Classification of Microplastics by a Wide-Field Fourier Transform Raman Microscope

**DOI:** 10.1021/acs.est.5c00165

**Published:** 2025-04-29

**Authors:** Benedetto Ardini, Lucia Pittura, Andrea Frontini, Maura Benedetti, Stefania Gorbi, Francesco Regoli, Giulio Cerullo, Gianluca Valentini, Cristian Manzoni

**Affiliations:** † Dipartimento di Fisica, Politecnico di Milano, Piazza Leonardo da Vinci 32, Milano20133, Italy; ‡ Dipartimento di Scienze della Vita e dell’Ambiente, Università Politecnica delle Marche, Ancona 60131, Italy; § NBFC, National Biodiversity Future Center, Palermo 90131, Italy; ¶ IFN-CNR, Istituto di Fotonica e Nanotecnologie, Piazza Leonardo da Vinci 32, Milano20133, Italy

**Keywords:** microplastics, Raman microscopy, hyperspectral microscopy, wide-field microscopy, Fourier transform spectroscopy, environmental microplastics

## Abstract

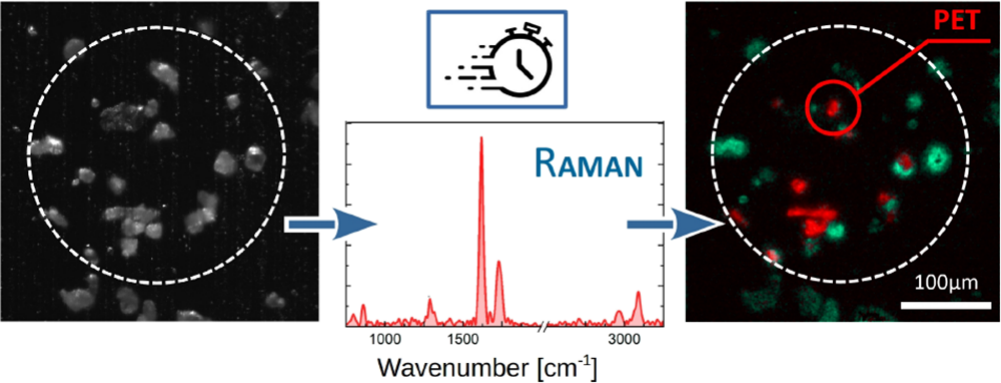

A number of applications require methods to detect with high spatial resolution and chemical specificity microplastics (MPs) extracted from different matrices. Here we introduce a wide-field hyperspectral Fourier transform Raman microscope for the rapid detection and identification of MPs. The instrument, based on a common-path birefringent interferometer, combines high spatial (∼1 μm) and spectral (∼23 cm^–1^) resolution with fast measurement times (∼15 min for a 100 kpixel image) and enables the suppression of sample fluorescence by a proper choice of the scan interval of the interferometer. After validating the instrument on MPs of commercial origin, we demonstrate its ability to detect MPs extracted from different matrices, by filtering seawater and pretreated gastrointestinal tracts of fish, and analyzing the MPs concentrated onto the filters. We expect that our microscope will enable high-quality, cost-effective, and rapid identification of MPs, fulfilling also the requirements of large-scale monitoring plans of different environmental matrices.

## Abstract 2

Novel methods and technologies are needed for efficient, time- and cost-effective identification and characterization of microplastics in different matrices. This study shows an innovative wide-field Raman microscope that meets these requirements.

## Introduction

The worldwide distribution of microplastics (MPs) in aquatic environments and their ingestion by virtually all species^1^ has given a huge input to monitoring programs and to the development of procedures for their detection and characterization. A plethora of studies have recently raised concerns for human health due to the ubiquitous contamination of MPs in air, water, and the food chain.^2−4^ For example, Marfella et al.^5^ found nanometer-sized MPs in atheromas of cardiovascular patients, who showed a greater risk of major complications compared to those with plastic-free plaques. This calls for a major effort to develop MP detection systems with better spatial resolution and specificity.

Most environmental matrices are processed to isolate MPs before their chemical identification. Although the type of treatment and the number of extraction steps depend on the sample, a vacuum filtration is generally needed to accumulate on filters the MPs contained in different typologies of matrices, such as water samples, supernatants obtained from a density gradient separation of sediments, or the digestates of biota samples.^6^ Suspected MPs on these filters are typically sorted out using optical microscopy, a low-cost and easy approach which provides information on the shape, size, and color of particles and represents a rational method for screening purposes or nonprofessionals.^7^ On the downside, the manual selection of suspected MPs requires time and effort when samples are not properly treated, and manpower to quantify particles, with a high risk of underestimation, especially due to the failure to detect small particles.^8^ Additionally, it does not provide information about the chemical composition of the MPs.

Compared with other pollutants, chemical analysis of MPs is generally complex and time-consuming, and different approaches are available, with both advantages and limitations. Thermo-analytical methods coupled with mass spectrometry, such as traditional pyrolysis gas chromatography mass spectrometry (Py-GC-MS) and its evolution, the thermal extraction desorption gas chromatography–mass spectrometry (TED-GC-MS)- are applied to detect plastic particles in different matrices without intensive sample purification or with no purification at all.^9^ These techniques enable the identification and mass-based quantification of synthetic polymers by relating their characteristic thermal degradation products to reference pyrograms and calibration curves of known virgin polymers. They can also be used to study volatile plastic-associated additives and contaminants, and detect very small particles, down to nanoplastics, as they are not dependent on the particle size, provided that their number is high enough to exceed the (mass-based) detection limit.^10^ The main drawback of thermo-analytical approaches is that they do not provide information on the number, size, and shape of MPs, although these are crucial to understand their bioavailability and possible toxicological effects.^11^ Furthermore, these methods are destructive, so that samples cannot be reanalyzed or analyzed by orthogonal techniques.^10^

To overcome these issues, different approaches coupling nondestructive imaging and spectroscopic analysis were proposed.^12−19^ Vibrational microscopy techniques, such as Fourier transform (FT) infrared (IR) and Raman microspectroscopy (μ-FTIR and μ-Raman), measure, for each point in the field of view (FOV), vibrational energy levels associated with the chemical bonds of a molecule, which have energies between 5000 and 500 cm^–1^. These methods have proven efficient for the nondestructive identification of MPs in field samples, providing spectral fingerprints for each type of plastic polymer based on its unique chemical structure.^20^ μ-FTIR instruments directly measure linear absorption of light in the mid-IR range, allowing a straightforward chemical identification; they exhibit excellent performances in terms of spectral resolution (∼1 cm^–1^), broadband detection of vibrational modes (2–25 μm spectral range, corresponding to 5000–500 cm^–1^)^21^ and short measurement times (∼10 min for ∼100 kpixels image);^14^ however, since they employ mid-IR wavelengths, they have low spatial resolution, thus preventing the identification of MPs smaller than ∼10–20 μm. Furthermore, they suffer from the high cost of the typically employed 2D pixelated IR detectors (focal plane arrays) based on mercury cadmium telluride. Recently, IR microscopy techniques alternative to μ-FTIR, such as Laser Direct Infrared (LDIR)^22,23^ and Optical Photothermal Infrared (O-PTIR)^24,25^ have been proposed to achieve faster MPs analysis and to enable the detection of particles smaller than 10 μm, respectively. However, despite these prominent advantages, these methods require quantum cascade lasers, which typically have a limited spectral tunability and make it difficult to detect vibrational modes above 3000 cm^–1^ and below 800 cm^–1^.^26,27^

μ-Raman techniques irradiate the sample with visible (e.g., 532 nm) or near IR (e.g., 785 nm) lasers; the frequency of the inelastically scattered light is shifted by an amount that corresponds to the vibrational modes of the system. The use of visible or near-IR radiation leads to a lower diffraction limit with respect to μ-FTIR, allowing the detection of MPs smaller than 10 μm.^28^ Typically, μ-Raman images are acquired by raster-scanning the FOV. Limitations of μ-Raman are the low quantum yield of the spontaneous Raman scattering process (10^–9^–10^–12^) and the low light throughput of the detection module, which make measurements very long (a few hundred milliseconds per pixel, several hours for a 100 kpixel image), and thus unsuitable for rapid MPs assessment, unless the spatial resolution is significantly reduced. In the last years Surface Enhanced Raman Scattering (SERS) has overcome this limit by significantly amplifying the Raman signal and achieving the detection of micro and even nanoplastics^29,30^ with reduced acquisition times; however, the substrates required for SERS represent a limitation to its real-world application, due to the need for close analyte contact and homogeneity, to degradation issues,^31^ and to the selective Raman enhancement only of particles with proper sizes and shapes.^30^ Another problem is the spectral overlap of the fluorescence background with the Raman signal, which can prevent MPs detection in highly fluorescent samples.

Hyperspectral microscopy (HSM) has recently emerged as a promising nondestructive, cost and time-effective technique for the identification of MPs without heavy sample manipulations.^32−37^ HSM combines optical microscopy with spectroscopy, providing spectroscopic information about each spatial point in the acquired 2D map of the measured sample. HSM is typically employed to detect transmission/reflection or fluorescence, which, however, has limited chemical specificity. Recently, some of the authors introduced a Raman Fourier-transform hyperspectral microscope (FT-HSM)^38^ that enables short measurement times (∼15 min for a 100 kpixel image) and the detection of fluorescence-free Raman spectra, with the same high spatial (∼1 μm) and spectral (∼23 cm^–1^) resolution typical of μ-Raman. These advantages arise from the fact that the new instrument integrates the capability of standard μ-Raman systems to detect and chemically identify small MPs with the advantages of FT spectroscopy that enables higher throughput, flexible spectral resolution, smart signal sampling, and parallel acquisition over a 2D FOV (wide-field approach).

This work aims to show the potential of such an innovative Raman FT-HSM as an effective methodology for the direct and rapid identification of MPs, including those isolated from complex environmental matrices. After introducing the operation principles of the microscope, we validated it on a series of commercial MPs to demonstrate its capability to discriminate and characterize different typologies of MPs. To further establish the applicability of the instrument for the characterization of MPs from different matrices, we filtered seawater samples containing spiked MPs. Finally, we considered fish gastrointestinal tracts, appropriately pretreated through conventional procedures, and demonstrated the use of Raman FT-HSM for chemical identification of the isolated MPs concentrated onto the filters.

## Materials and Methods

### Raman FT-HSM Overview and Operating Principles

**Figure 1:**
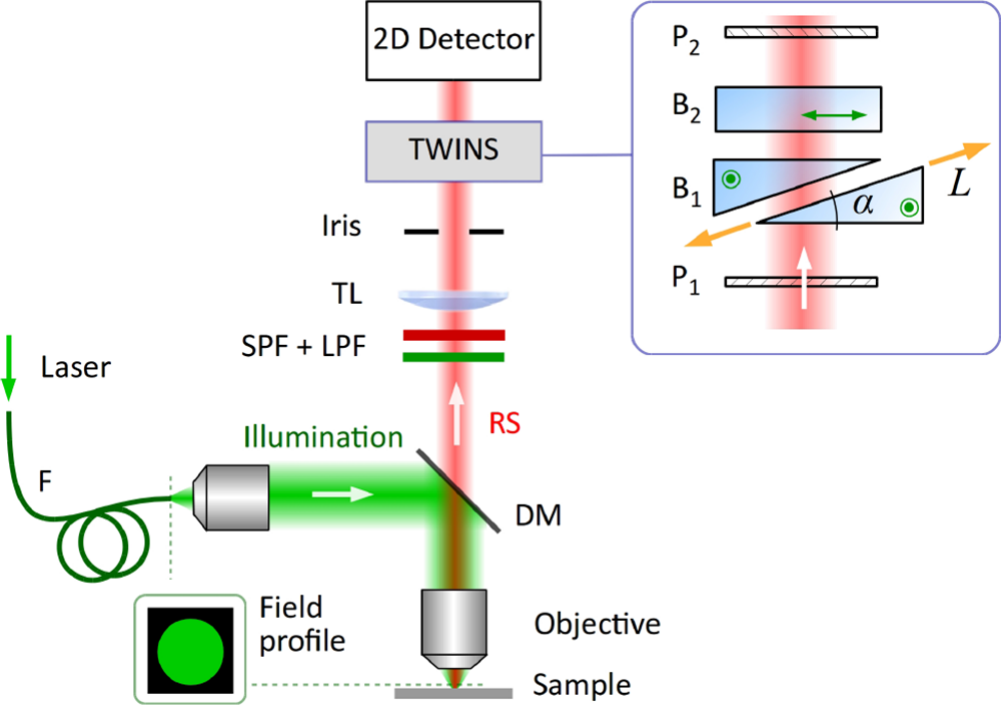
Scheme of the FT-HSM setup. P_1/2_: linear polarizers; B_1/2_: birefringent blocks, whose optical axes are represented by green circles and double-arrow; F: multimode optical fiber; DM: dichroic mirror; RS: Raman Scattering; TL: tube lens; SPF: short-pass filter for the suppression of fluorescence; LPF: long-pass filter for rejection of illumination light.

The FT-HSM, whose scheme is reported in Figure 1, consists of a commercial microscope (Leica DMRBE) coupled to an ultrastable birefringent interferometer, the Translating-Wedge-Based Identical Pulses eNcoding System (TWINS). The interferometer is located between the tube lens (TL) and a silicon CCD camera with 1002 × 1004 pixels, which also enables hardware pixel binning to increase the signal-to-noise ratio (SNR). TWINS^39^ splits the incoming light wavefront into two collinear delayed replicas with a stability of their relative delay better than 1/360 of the optical cycle at 600 nm wavelength: the intensity of the interfering replicas acquired on each detector pixel as a function of delay constitutes the measured interferogram. Since the acquisition occurs in parallel for all the pixels of the camera, this is true wide-field imaging, which is one of the main advantages of our system. We use a TWINS based on two blocks of the birefringent crystal YVO_4_ (B_1_ and B_2_): B_1_ is shaped into two wedges (apex angle α = 10°, transverse size 30 mm), one of which is laterally translated by a linear motorized stage (minimum incremental motion: 0.02 μm) that scans the relative delay of the replicas.

The excitation light is generated by a frequency-doubled Nd:YAG laser at λ = 532 nm (NPS, Bright Solutions, Pavia, Italy), which is coupled to the microscope through a 400-μm-core multimode optical fiber, reflected by a dichroic mirror (DM), and sent through the microscope objective. This last can be changed according to the desired magnification: in this work, we used 10× (NA 0.30), 20× (NA 0.40), and 100× (NA 0.75) objectives. A relay system is used to image the tip of the fiber on the object plane, providing a uniform (top-hat) illumination spot (82*%* of the FOV diameter): the Raman signal propagates through a 532 nm long-pass filter (LPF), rejecting the residual illumination light, a 650 nm short-pass filter (SPF), removing fluorescence light beyond large Raman shifts (>3412 cm^–1^), and then imaged on the 2D detector. Further technical details on the microscope components are listed in the SI.

The measurements are recorded by acquiring on the 2D detector one frame for each delay set by the TWINS. The Raman signal is often overlapped with a strong autofluorescence background. However, as briefly mentioned in the introduction, the FT-HSM approach has the unique advantage of separating the two contributions.^38^ This arises from the fact that the interferogram produced by the broadband fluorescence is characterized by a few oscillations at early delays, while the narrowband Raman field has long-lasting oscillations at large delays. In practice, this feature enables us to obtain either the fluorescence or the Raman signal by simply selecting the scan range. For this reason, to suppress the spectral features of the fluorescence signal, the starting point of the scan is set at 34 fs (see the SI for details).

One last advantage of the FT-HSM technique is the flexibility in the choice of the spectral resolution, which depends on the length of the delay interval. In the following, we will employ two scan intervals (detailed in Table 1): (i) a long scan providing the highest spectral resolution allowed by our FT-HSM (23 cm^–1^) at the expense of longer measurement times and (ii) a short scan providing ∼4.5-times faster measurements but with lower spectral resolution (105 cm^–1^). The acquired time-domain interferometric raw data are first preprocessed to improve the SNR through singular value decomposition (SVD) denoising,^40^ and then, the FT operation is applied on each pixel’s interferogram to retrieve the related spectrum. The spatial maps of the different Raman signatures in the imaged FOV are then obtained with endmember-extraction algorithms (MCR^41^ or N-FINDR^42^) on the spectral data set. All the preprocessing steps and the data analysis are performed with in-house software.

**Table 1:** TWINS Scans for the Acquisition of the Interferograms Described in this Study^a^

	low spectral resolution	high spectral resolution
scan range (fs)	34 → 585	34 → 2524
spectral resolution (cm^–1^)	105	23
number of acquired frames	678	3064

a Scan range and step refer to a wave with a 600 nm wavelength.

### Validation of Raman FT-HSM with Commercial MPs

The capability of Raman FT-HSM to identify and characterize MPs was initially tested using commercial MPs. Various particles, differing in polymer types, shapes, and sizes, were selected and obtained from different Italian companies: (i) fragments of polypropylene (PP, 50–1000 μm), polyvinyl chloride (PVC, 30–230 μm), poly(vinyl alcohol) (PVA, 50–150 μm), polyethylene (PE, 20–500 μm), polystyrene (PS, 50–1000 μm), (ii) fibers of polyester (PET, 618 ± 367 μm in length and 13 ± 1 μm in diameter) and polyamide (PA, 566 ± 500 μm in length and 11 ± 1 μm in diameter); (iii) microbeads of poly(methyl methacrylate) (PMMA) and PS, both 3 μm in diameter. The analyses were performed on both individual typologies and a combination of MPs, which were deposited on an aluminum substrate that exhibits no Raman signal. Short scans were taken, and different sets of acquisition parameters, such as binning, irradiance, and integration time, were tested to assess the system’s performance and versatility.

### Validation of Raman FT-HSM with Micronized Beached Plastic

The Raman FT-HSM was also tested to assess its ability to analyze noncommercial MPs. For this purpose, MPs were obtained by micronizing plastic debris collected during beach cleaning activities carried out in the framework of an international project (RESPONSE JPI Oceans) and already used for ecotoxicological studies.^43,44^ Briefly, PET bottles were micronized to obtain MPs (hereafter termed PET-bottles-MPs) with sizes smaller than 20 μm. PET-bottles-MPs were placed on an inert aluminum substrate, as in the case of commercial MPs. For this acquisition, we used the 20× microscope objective. We initially acquired a measurement with low spectral resolution to ascertain the presence of MPs in the FOV (see Figure S1 in the SI) before running the high-resolution scan. The laser power on the sample was 230 mW (irradiance: 270 W/cm^2^); the signal was further increased by applying a 2 × 2 hardware binning, resulting in a 251.5 kpixel image (the corresponding pixel sampling is ∼0.8 μm, comparable to the diffraction-limited spatial resolution). The exposure time per frame was set to ∼450 ms, resulting in a total delay-scan time of 36 min.

A second measurement run characterized a mixture of MPs in the size range of 20–50 μm composed of PE, PET, PP, PS, and PVC. The mixture, hereafter referred to as MP-mix, was obtained by micronizing beached, hard-plastic containers, and it was added (spiked) to prefiltered 1 L seawater before undergoing a conventional vacuum filtration procedure using a membrane filter with 8 μm pore size made of mixed cellulose ester (MCE); we opted for this type of membrane because MCE has a lower Raman signal compared to nylon and cellulose acetate (CA) (see Figure S2 in the SI). As in the case of PET bottles, we used the 20× microscope objective with ∼270-W/cm^2^ laser irradiance, and we performed a preliminary measurement with low spectral resolution (see Figure S4 in the SI) to ascertain the presence of MPs in the FOV. The subsequent high-resolution measurement (spectral resolution: 23 cm^–1^) enabled us to resolve possible spectral deviations from tabulated polymers’ spectra, typically observed in environmental MPs. A 4 × 4 binning was applied, resulting in a 62.5 kpixel image (pixel sampling: ∼1.6 μm) and the exposure time per frame was set to 200 ms, resulting in a total measurement time of 19 min.

### Validation of Raman FT-HSM for MPs Extracted from Biota

For this phase, specimens of mackerel, *Trachurus trachurus* (*n* = 8) were retrieved from fishermen in the Adriatic Sea, and the gastrointestinal tracts were processed to extract MPs according to procedures already validated for biotic matrices.^45,46^ Briefly, the tissues were treated with a 10*%* potassium hydroxide (KOH) solution (incubation at 50 °C)^47^ to completely digest the organic matter and then vacuum filtered on MCE membranes (8 μm pore size). Samples were analyzed by Raman FT-HSM with a 10× objective. To further validate the accuracy of the results, the filters were also examined under the stereomicroscope and the identified particles were analyzed with μATR-FTIR spectroscopy (Spotlight 200i FTIR microscope system, PerkinElmer; see IR spectra in Figure S9 of SI), widely established as an effective method for MPs characterization.^6,20^

To prevent airborne and cross-contamination, several precautions were implemented during the filtration processes, which were already widely reported in refs (45−47) and which are detailed in the SI.

## Results and Discussion

### Validation of Raman FT-HSM with Commercial MPs

The measurement of commercial MPs allowed us to assess the instrument performances and versatility to identify MPs with different shapes, sizes, and polymeric composition and to verify their capability to resolve particles smaller than 10 μm, achieving spatial resolution well below the limit of μ-FTIR.

**Figure 2:**
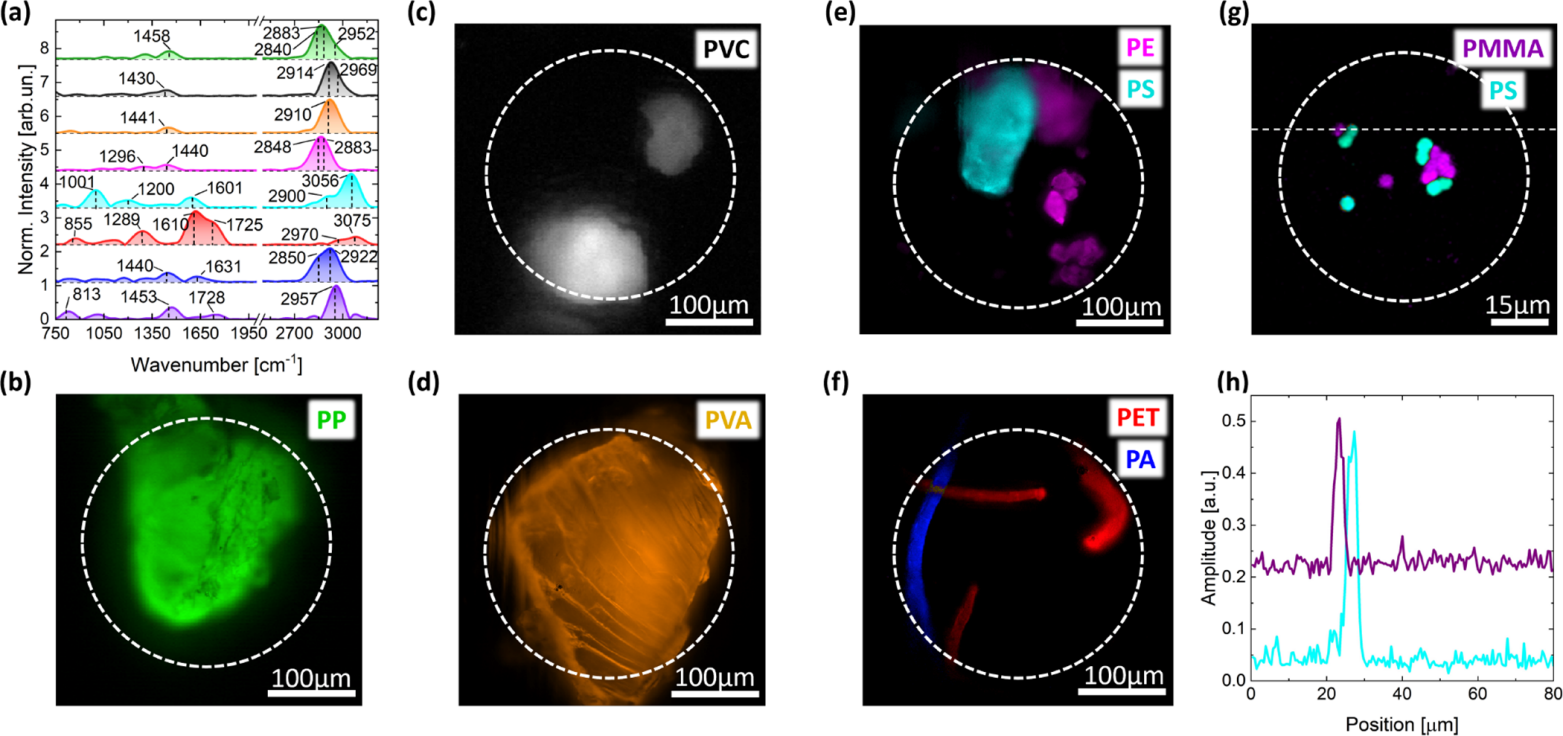
Wide-field Raman microscopy of commercial MPs. (a) Spectra obtained from endmember-extraction analysis on separated measurements in panels (b–g). Dashed vertical lines and related values correspond to the main Raman peaks reported in the literature for the measured MPs species. (b–g) Spatial maps of commercial MPs, each one showing in color the pixels containing the corresponding endmember spectrum in panel (a). The white dashed circumference identifies the illuminated area of the sample. (h) Spatial profiles of PMMA and PS maps in the horizontal cut are outlined in panel (g).

As shown in Figure 2, our FT-HSM system effectively detects the Raman signal from MPs and identifies their characteristics in the acquired FOV on the basis of their spectral signature. Even from the fast scan (low spectral resolution, Table 1), the retrieved bands (Figure 2a) match the main Raman peaks reported in the literature,^48−51^ and are sufficient to preliminarily discriminate the polymers. This result demonstrates the advantage of Raman FT-HSM over standard μ-Raman systems in reducing the total measurement time by sacrificing the spectral resolution rather than the spatial one: such an approach is crucial when large FOVs have to be rapidly investigated and differentiation of MPs from nonpolymeric particles is more important than a precise classification of their Raman signature (see comparison of MPs’ spectra with those of nonpolymeric species in Figure S4 of the SI).

The measurement parameters, as well as the microscope objective, were tailored to the different types of MPs, as summarized in Table 2. Large fragments characterized by uneven not-flat surface, as in the case of PP (Figure 2b) and PVC (Figure 2c), have many out-of-focus contributions: hence for these measurements we opted to relax the spatial resolution in favor of a high CCD binning to increase the SNR, rather than delivering high laser irradiance on the sample. In fact, the dissipation of the internally generated photothermal load toward the environment is typically less effective in large samples than in small fragments. Vice versa, in the case of small or flat MPs (Figure 2d–f), where the identification of tiny features is important, we used low CCD binning to have high spatial resolution and we increased the laser irradiance to improve SNR. The measurement of plastic microbeads shown in Figure 2g is an exception as the large magnification provided by the use of the 100× objective allowed a sufficient pixel sampling even with a 6 × 6 binning. This last measurement and the ones shown in panels (e) and (f) of Figure 2 show the capability of our Raman FT-HSM to effectively separate different polymer species in the same FOV and to detect MPs as small as 3 μm.

**Table 2:** Acquisition Parameters for the Measurement of Commercial MPs

	PP	PVC	PVA	PE & PS	PET & PA	PMMA & PS
objective	20×	20×	20×	20×	20×	100×
CCD binning	3 × 3	5 × 5	2 × 2	2 × 2	2 × 2	6 × 6
pixel sampling (μm)	∼1.2	∼2	∼0.8	∼0.8	∼0.8	∼0.5
number of kpixels	111.6	40.0	251.5	251.5	251.5	27.9
irradiance on sample (W/cm^2^)	∼295	∼295	∼530	∼530	∼530	∼1865
total acquisition time (min)	4	3	32	14	14	9

### Validation of Raman FT-HSM with Micronized Beached Plastics

**Figure 3:**
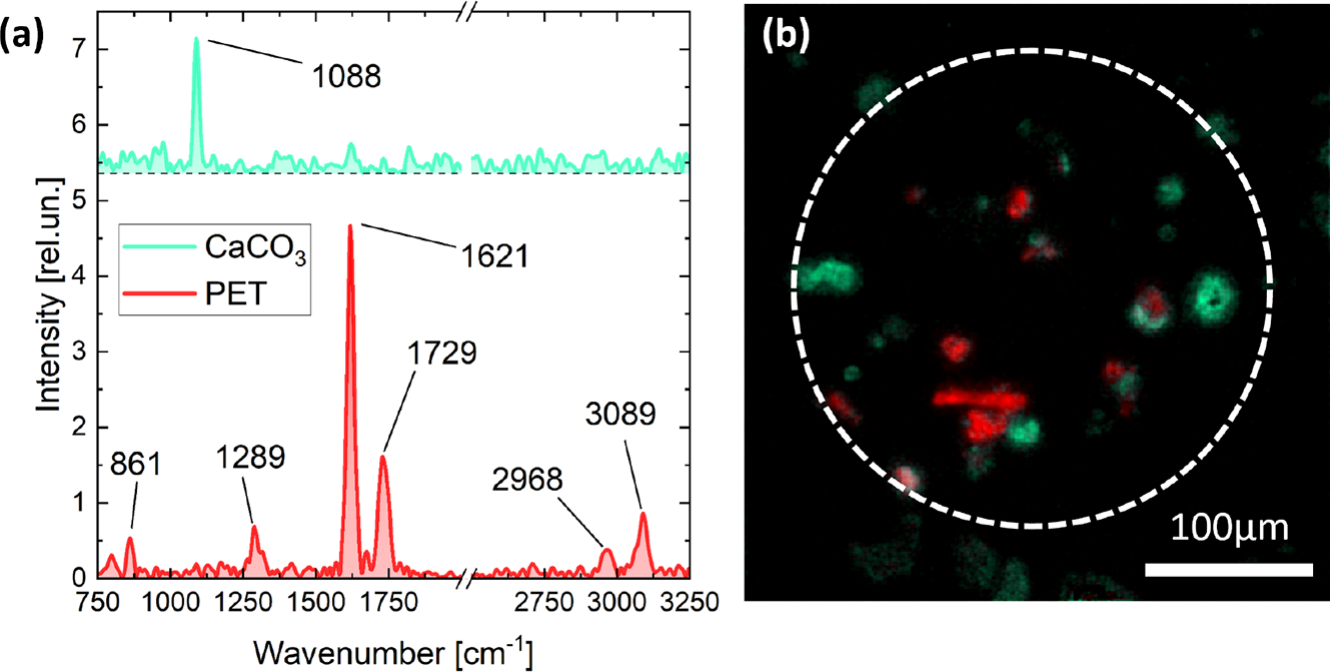
Wide-field Raman microscopy of PET-bottle MPs. (a) Spectra selected in regions of interest in CaCO_3_ and PET. (b) Map obtained from N-FINDR analysis on spectral hypercube. The white dashed circumference identifies the illuminated area of the sample.

Figure 3 shows the results of the analysis of the PET-bottles-MPs over an aluminum substrate: in addition to MPs, we also detected calcium carbonate (CaCO_3_) particles, identified by their typical band at 1088 cm^–1^ (symmetric stretching vibration, ν_1_)^52,53^.^54−56^ The presence of such material is expected since it represents one of the most common constituents of sand, mostly derived from calcareous shells and skeletons of dead marine organisms.^57^ Moreover, we have to consider that no pretreatment was applied to either the beached plastic or the PET-bottles-MPs to remove inorganic material (e.g., by washing, density separation, etc).

The absence of overlapping peaks demonstrates the capability of the instrument to effectively distinguish synthetic polymers from nonpolymeric materials within the same matrix and without sample preparation. This is an important feature for a reliable determination of MPs in environmental samples, since most of the missed or erroneous identifications through vibrational spectroscopic techniques are related to background noise and fluorescence of matter naturally associated with the surface of plastic items.^58^ This especially occurs if the material cannot be eliminated by chemical digestion (i.e., minerals)^59^ or when digestion treatments are not applied to the sample, as is the case with beach sand, which is considered to be a matrix “cleaner” than subtidal sediment due to the lower content of organic matter.^57^

Fluorescence due to the material itself or plastic additives (e.g., pigments) can affect the Raman scattering of MP samples, introducing a strong background. This was highlighted by Peñalver et al.^60^ and González-Fuenzalida et al.,^61^ comparing Raman spectra of pristine and recycled PET containers. The latter showed, in fact, a higher fluorescence that was ascribed to impurities characterizing recycled materials. For the same reason, Marku and Chatzhitheodoridis^62^ could not resolve the Raman bands of PE for colored and recycled PE-granules. High background signals from the pigments were also detected by Liu et al.,^63^ who reported the Raman spectra of colored meso- and microplastics, made of different polymers, with serious interference. The hyperspectral fluorescence map on the same FOV as reported in Figure 3 (see Figure S5 in the SI) shows that the same PET particles have different fluorescence spectra, which are probably related to dyes or impurities on the MPs. Despite this, thanks to the capability to separate Raman and fluorescence spectra offered by our FT-HSM, it was possible to resolve Raman spectra of PET, corroborating the capability of the device to identify MP polymers even when they are contaminated by strongly fluorescent materials.

The clear detection of PET-bottles-MPs among CaCO_3_ particles was possible even with the low-spectral-resolution scan (see Figure S1 in the SI). This confirms that the advantage of speeding up measurements without reducing the spatial resolution, which we tested on commercial MPs, can also contribute to the fast and effective detection of small MPs in environmental samples. Such a unique characteristic of FT-HSM is of paramount importance since a large number of samples must be investigated for an adequate environmental assessment. The map of the PET-bottles-MPs sample (Figure 3b) also confirms the ability of the instrument to detect MPs smaller than 20 μm, not identifiable with μ-FTIR platforms, which has been previously validated by detecting microbeads of PMMA and PS (Figure 2g,h).

Based on the outcomes of the analysis of PET-bottles-MPs, we propose Raman FT-HSM as a promising method for the direct identification of MPs in beach sand samples, also enabling the characterization of the smaller fragments, which are likely neglected during visual sorting after density separation.^36^ Also, we demonstrate the ability to adapt the acquisition parameters to the specific problem (i.e., preliminary screening to rapidly recognize plastic from nonplastic material, or precise study of the spectral features of MPs), guaranteeing in any case the effectiveness of results (i.e., MPs identification).

In this study, we demonstrate that cellulose-based filters (i.e., CA and MCE ones) have weaker Raman peaks than nylon filters (Figure S2 in the SI), in contrast with what was observed by Piarulli et al.^32^ by near-infrared hyperspectral imaging while detecting MPs in digested tissues. Therefore, to select the most suitable filter material, it is necessary to also take into account the specific spectroscopic technique. Piarulli et al.^32^ highlighted that cellulose filters maintained the original size and shape of MPs because of the lower aggregation of particles after filtration compared to nylon supports, as a consequence of the different properties of the material itself. This is an additional aspect that should drive the choice of the filter type since it affects the subsequent characterization of the particles. Furthermore, comparing Raman spectra of CA and MCE filters (see Figure S2 in the SI), it is clear that the latter represents the best option for MPs measurements with our Raman FT-HSM system. This consideration led us to use MCE filters for the detection of MPs after the filtration of spiked seawater samples, MP-mix.

**Figure 4:**
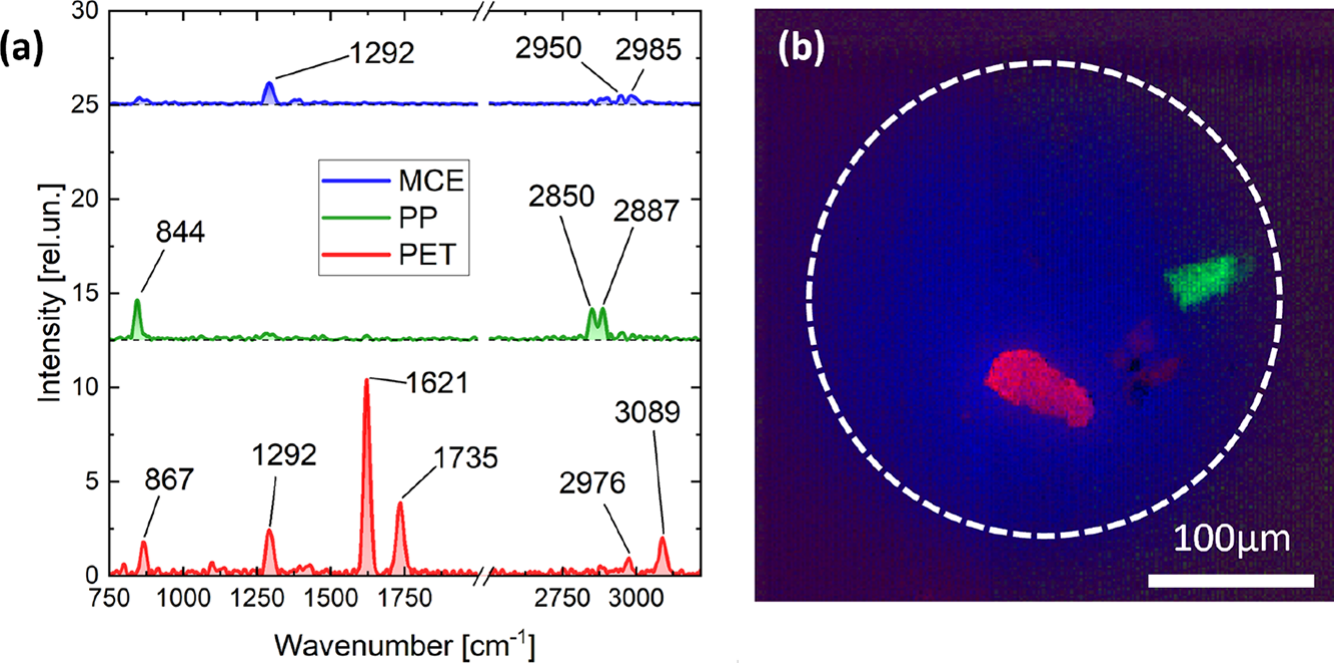
Wide-field Raman microscopy of the MP-mix on the MCE filter. (a) Endmember spectra retrieved by N-FINDR analysis. (b) Composite map of the endmember spectra shown in panel (a). The white dashed circumference identifies the illuminated area of the sample.

Figure 4 shows the result of the characterization of MP-mix samples, which clearly identifies MPs made of PP and PET, on the basis of their characteristic Raman peaks,^51,64−68^ and separates them from the MCE background despite the presence of the peak at 1292 cm^–1^ in the PET-MP spectrum that is common with the MCE filter. Figure S7 shows the measurement of a filter obtained after vacuum filtration of a seawater sample without spiked MPs (i.e., a blank sample): we obtained a uniform Raman signature of the MCE in the acquired FOV, which demonstrates the absence of any foreign particles and therefore possible external or cross contamination.

Although MCE filters were considered the best choice for this analysis, it is necessary to take into account the possibility of finding this additional peak in the MPs spectra. The Raman band at 844 cm^–1^ of the PP spectrum was attributed to a chromate-based pigment,^69−71^ highlighting again that in our instrument, the interpretation of peaks is not hindered by the possible presence of a fluorescence background associated with dye additives, because any spectral feature of fluorescence is automatically removed during the measurement phase. This interpretation is supported by the absence of this specific peak in the uncolored commercial PP (Figure 2). However, we cannot exclude that the Raman band at 844 cm^–1^ is instead linked to the polymer, being diagnostic of the amorphous structure of syndiotactic PP compared to highly crystalline isotactic PP.^72^ In fact, diagnostic peaks of a certain plastic polymer can also vary in intensity, width, or position due to configurational and conformational variants of its basic structure (i.e., crystalline or amorphous state and trans or gauche arrangement).^73^

### Validation of Raman FT-HSM for MPs Extracted from Biota

A current research challenge in environmental science is to simplify the analysis of MPs extracted from different matrices by introducing new approaches for their morphological and polymeric characterization to achieve high-quality, cost-effective, and rapid identification. This is crucial, in particular, when routine and large-scale monitoring plans need to be implemented to assess the environmental status of ecosystems, such as in the context of the marine strategy framework directive (MSFD, 2008/56/EC), or in support of a valid risk assessment for human health, as required by the recently reviewed drinking water directive (Directive (EU) 2020/2184).^46,74−77^

In this context, the fast acquisition of our Raman FT-HSM enabled by the wide-field approach and by the possibility to adjust the spectral resolution greatly contributes to the identification of unknown MPs extracted from field-collected organisms.

**Figure 5:**
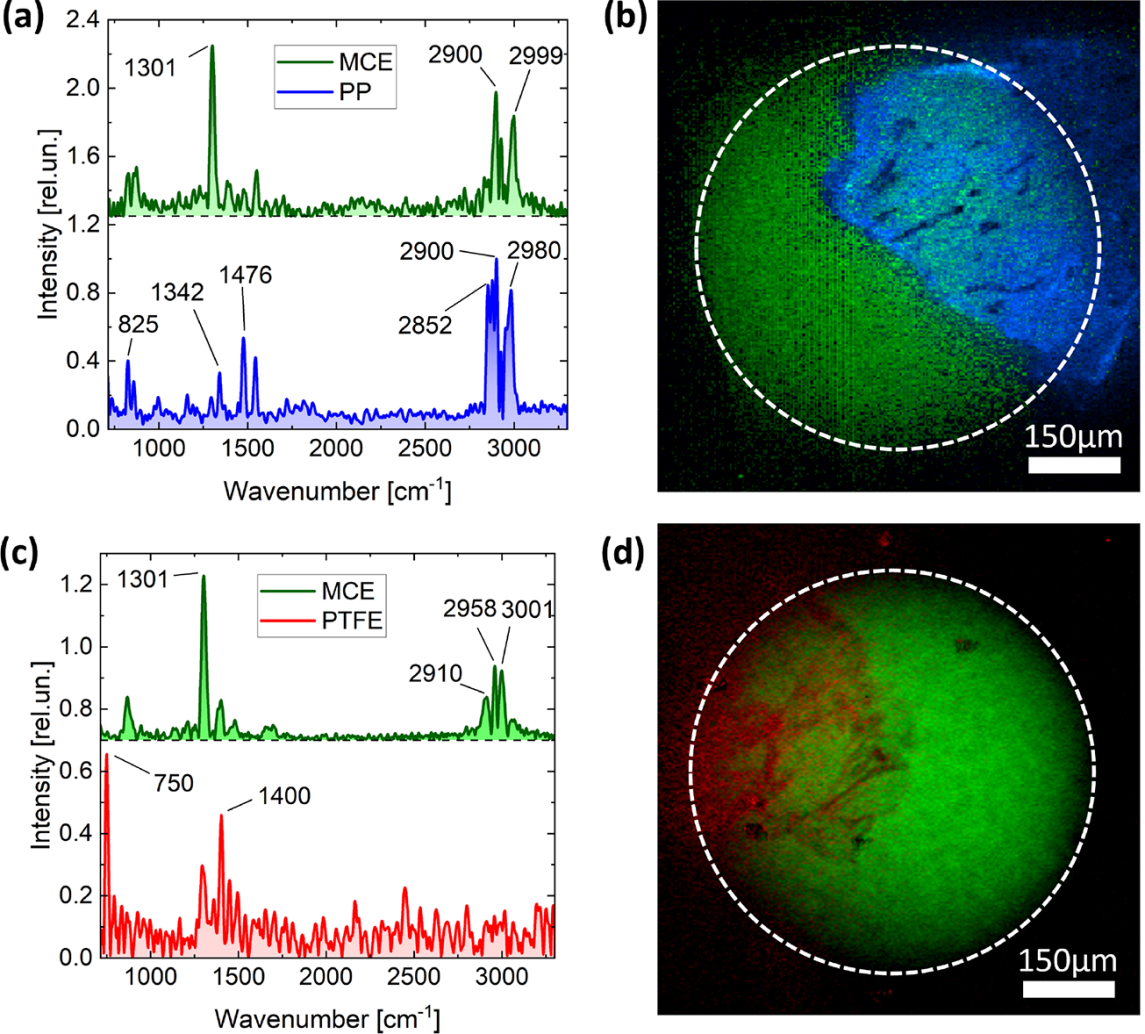
Wide-field Raman microscopy of MPs extracted from the gastrointestinal tracts of mackerel *Trachurus trachurus* (a–d). (a,c) Endmember spectra retrieved by MCR analysis. (b,d) Composite map of the endmember spectra shown in panels (a) and (c) respectively. The white dashed circumference identifies the illuminated area of the sample. (a,b) Total acquisition time: 13 min; laser irradiance on the sample: 53 W/cm^2^; CCD binning: 4 × 4; number of pixels: 62.5 kpixels; pixel sampling: 3.2 μm. (c,d): 50 min; laser irradiance on the sample: 44 W/cm^2^; CCD binning: 4 × 4; number of kpixels: 62.5; pixel sampling: 3.2 μm.

The ability of the Raman FT-HSM to characterize MPs isolated from the biotic matrices was demonstrated on the gastrointestinal tracts of the fish *Trachurus trachurus*; the technology successfully enabled morphological and chemical MP identification after their isolation onto a filter. In particular, the analyses of filters obtained from the processing of the fish gastrointestinal tracts highlighted the presence of MPs in 2 samples out of 8. We detected MPs in 25*%* of collected organisms, a percentage in line with other studies on the same species.^46,78,79^ Specifically, two MPs were found (one particle per positive individual), one made of PP and one of polytetrafluoroethylene (PTFE) (Figure 5), based on the characteristic Raman peaks of polymers.^80,81^ Results were also corroborated by the absence of contamination by any items in the corresponding blank sample. The polymeric origin of the extracted MPs was also confirmed by μFT-IR spectroscopy (see SI).

In conclusion, we introduced wide-field Raman FT-HSM for the rapid detection and identification of MPs. The instrument is based on the TWINS common-path birefringent interferometer and combines (i) high spatial resolution (∼1 μm) in a large FOV (up to ∼400 μm); (ii) spectral resolution down to 23 cm^–1^, and (iii) fast measurement times (∼15 min for 100k pixels image).

The versatility in decreasing measurement time (at least by a factor of 4.5 with low-spectral-resolution scans) allows for a rapid screening of MPs. This result is obtained without sacrificing spatial resolution, unlike in traditional Raman systems. Furthermore, the time-domain FT approach enables the suppression of sample fluorescence by a proper choice of the scan interval of the interferometer. After validating the instrument on commercial MPs, we demonstrated its ability to distinguish MPs in different matrices by filtering spiked seawater and analyzing MPs extracted from biotic matrices. Moreover, our FT-HSM approach has proven capable of efficient detection of MPs in nonpretreated samples on filters, since it is unaffected by the fluorescence of chemical species from biological/mineral components and/or additive dyes.

A present limitation of our approach is photothermal load due to absorption of pump light by the sample. This is because, contrary to point-like excitation in standard μ-Raman, the wide illuminated area allows only a one-dimensional thermal dissipation through the sample’s bulk. We expect that it will be possible to increase the laser irradiance by applying proper heat dissipation strategies, for instance, by performing measurements in water immersion.

## Supporting Information Available

Working principle of the FT-HSM, spatial and spectral resolution of the microscope, PS/PMMA beads: sample preparation, low-spectral-resolution Raman of PET-bottle MPs sample and mixed environmental species, filter selection, comparison of MPs with other species, fluorescence measurement on PET-bottle MPs sample, Raman measurement of the MCE filter obtained after vacuum filtration (blank sample), and comparison with standard Raman spectroscopy (PDF)
