# Supplementary material for: Fast Detection and Classification of Microplastics by a Wide-Field Fourier Transform Raman Microscope
Source: Environ Sci Technol. 2025 Apr 29;59(18):9255–64. doi: 10.1021/acs.est.5c00165 (PMC12080255; doi:10.1021/acs.est.5c00165)
Supplement: Supplementary file 1 — es5c00165_si_001.pdf [file es5c00165_si_001.pdf]

Fast detection and classification of microplastics by a wide-field Fourier transform Raman microscope: Supporting Information

Benedetto Ardini,[†,‖] Lucia Pittura,[‡,¶,‖] Andrea Frontini,[‡] Maura Benedetti,[‡,¶] Stefania Gorbi,[‡,¶] Francesco Regoli,[‡,¶] Giulio Cerullo,[†,§] Gianluca Valentini,[†,§] and Cristian Manzoni[*,§]

†*Dipartimento di Fisica, Politecnico di Milano, Piazza Leonardo da Vinci 32, Milano, 20133, Italy*

‡*Dipartimento di Scienze della Vita e dell'Ambiente, Università Politecnica delle Marche, Ancona, 60131, Italy*

¶*NBFC, National Biodiversity Future Center, Palermo, 90131, Italy*

§*Istituto di Fotonica e Nanotecnologie, IFN-CNR, Piazza Leonardo da Vinci 32, Milano, 20133, Italy*

‖*B.A. and L.P. contributed equally to this work*

E-mail: cristianangelo.manzoni@cnr.it;cristian.manzoni@polimi.it

- Number of pages: 15
- Number of Figures: 9
- Number of tables: 2

Working principle of the FT-HSM

FT spectroscopy, which measures a time-domain interferogram, offers a great advantage over dispersive spectrometers because it provides access to different features of the spectrum by tailoring the scanning strategy. One advantage is the ability to separate fluorescence from Raman signals.

To illustrate the method, we recall two properties of the FT:

(a) the bandwidth of an optical signal is inversely proportional to the duration of the corresponding interferogram. As a consequence, the interferogram produced by a broadband signal has significantly fewer optical cycles than the one of a narrowband field;

(b) the FT is a linear operator, hence the interferogram of a linear combination of uncorrelated spectra is the linear superposition of the individual interferograms.

These properties can be used to decompose a spectrum into its broadband and narrowband components, according to the approach illustrated in Fig. S1. Panel (a) shows a spectrum consisting of narrow features (*e.g.* Raman peaks) overlapped to a broad back-ground (*e.g.* fluorescence). The corresponding interferogram is given in panel (b). It is characterized by large oscillations at early delays and tiny signals at long delays, whereas its average value C is proportional to the integrated spectrum. The signal at short delays corresponds mostly to the broad spectral features, while the oscillations at long delays are only due to the narrowband components. One can therefore separate these signals by taking the FT of the symmetric early-delay interferogram (green dashed box) or of the long-lived oscillation. In the latter case, we only consider the portion of the interferogram at large positive delays (blue dashed window). Panel (c) shows the resulting spectra, that corre-spond to the broadband and narrowband components of the initial spectrum, which are now completely separated. This method allows the extraction of fluorescence-free Raman spectra and offers an additional advantage: since the scan does not include the strongly oscillating interferogram at early delays, the long-lived oscillations

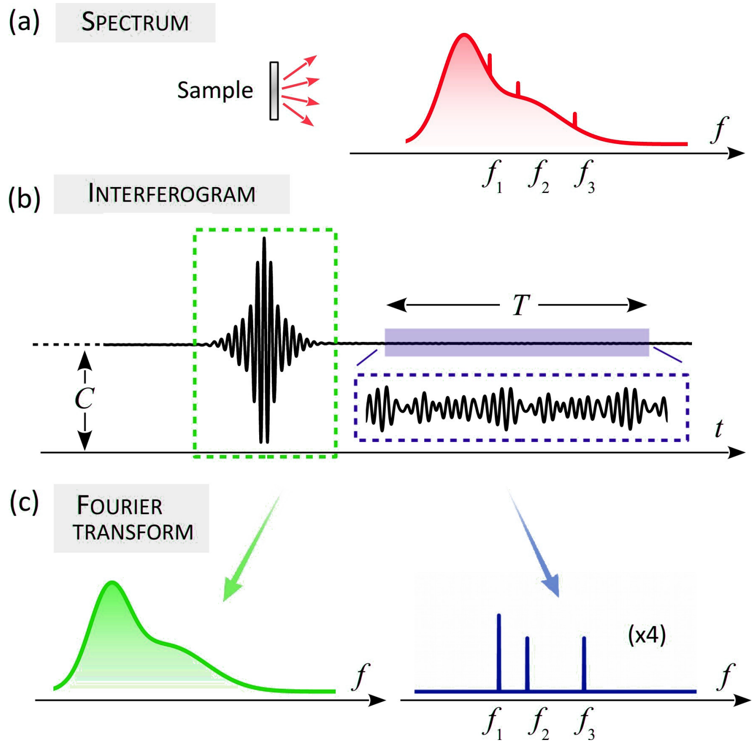

Figure S1: Schematic representation of the FT approach in the case of a multimode spec-trum (a), characterized by both broadband (*e.g.* fluorescence) and narrowband (*e.g.* Raman peaks) features. (b) The corresponding interferogram, characterized by the overlap of promi-nent few-cycle oscillations at early delays and weaker signals lasting for a large number of cycles. (c) The application of the FT to the early delay fraction of the interferogram conveys the broadband, slowly varying spectrum; the long-lasting oscillations at longer delays give rise to narrowband spectra.

can be obtained with a better dynamic range, because the signal can be integrated for a longer time before saturating the detector.

During the acquisition, the scan is defined by the initial (T_1) and final (T_2) delays of the interferogram. The proper choice of T_1 and T_2 enables selecting the fluorescence or the Raman signals: it is on this unique advantage that our multimodal microscope is based.

It must be noted that in practice this method is advantageous only in an FT-based detection system, as in the case of our FT-HSM. Conversely, the application of the same time-filtering approach to the numerical FT of a measured frequency-domain spectrum would lead to a severe degradation of the retrieved peaks, because of noise.

The delays T_1 and T_2 also determine the *scan range* $T = |T_2 - T_1|$, which is inversely proportional to the spectral resolution of the interferometer; T is hence chosen according to the resolution required by the application.

Here we also provide the detailed characteristics of the equipment used for the measurements reported in the main text:

- Objectives:

  10 ×: Leica®PL FLUOTAR, NA 0.30

  20 ×: Leica®PL FLUOTAR, NA 0.40

  100 ×: Leica®PL FLUOTAR, NA 0.75

- Detector: CCD camera, Luca R, Andor (Belfast, Northern Ireland), size 8×8 mm^2, 1002×1004 pixels, 14-bit depth, spectral sensitivity from 400 nm to 1100 nm

- Mirrors and filters:

  DM : Dichroic mirror, Semrock Di02-R532-25x36

  LPF : 532-nm long-pass filter, Semrock LP03-532RU-25

  SPF : 650-nm short-pass filter, Thorlabs ESH0650

- Translation stage: Physik Instrumente M-112.12S1, 119 Karlsruhe, Germany. The delay is sampled in steps of 0.813 fs, thus satisfying the Nyquist-Shannon criterion (step < 0.887 fs) to properly sample the signal at the highest frequency, which in our case is the 532-nm pump wavelength.

Spatial and spectral resolution of the microscope

The *spectral resolution* of the hyperspectral microscope is estimated by measuring the interferogram of a monochromatic wave (*e.g.* the Raman pump), and calculating the width of its spectral peak after FT. Such width depends on the scan length and on the apodization function applied to the interferogram before taking the FT. The spectral resolution for the standard scans used for the paper are reported in Table S1.

The *spatial resolution*, on the other hand, is given by two factors: the diffraction (or Abbe) limit and the pixel sampling.

The diffraction limit is given by the optics of the microscope (mostly the objective) and the wavelength of the measured light. In the visible spectral range, the spatial resolution of our microscope is of the order of 1 μm. This number must be compared with the pixel sampling, which depends on the pixel size and the binning: it represents the smallest detail captured by each pixel. Values of the pixel sampling are reported in Table S2: in most of the measurements reported in the paper, the spatial resolution is posed by the diffraction limit.

PS/PMMA beads: sample preparation

50 μL of polystyrene (PS)microparticles (72986-5ML-F, MerckKGaA, Darmstadt, Germany) and 50 μL of poly(methyl methacrylate) (PMMA) microparticles (90515-5ML-F, Merck KGaA, Darmstadt, Germany), of 3 μm in diameter, were diluited in 600 μL of water. 5 μL of this aqueous suspension were deposited on an aluminum substrate and air dried for 30 minutes.

Low-resolution Raman of PET-bottle MPs sample

Before acquiring the high-resolution measurements of environmental PET-bottle MPs (Figure 3 in the main text) we performed a preliminary low-resolution acquisition (spectral resolution 105 cm^{-1}) of the same FOV. As it is clear from Figure S2, this low-resolution measurement is enough to have a first identification of MPs in the FOV.

Contamination control

To prevent air-borne and cross-contamination, several precautions were implemented during the filtration processes. These quality assurance/quality control procedures also included control samples (*i.e.* blank samples) and more specifically: the blank sample for the spiking experiment consisted of a 100-ml glass beaker containing pre-filtered seawater that was left open on the workbench during the addition of MPs. It was then filtered and analyzed in the same way as the spiked samples. On the other hand, the blank sample related to the analysis of MPs in the biota consisted of a 100-ml glass beaker containing pre-filtered 10% KOH solution left open on the workbench starting from dissection and then filtered and analyzed in the same manner as the field samples.

Filter selection

The selection of the best filter has been performed by measuring each filter with a low-resolution FT-HSM acquisition. The measurement parameters are listed in Table S1. By comparing their spectra (Figure S3) it is clear that the MCE filter is the most suitable for MPs measurements as it shows the lowest Raman peaks intensity.

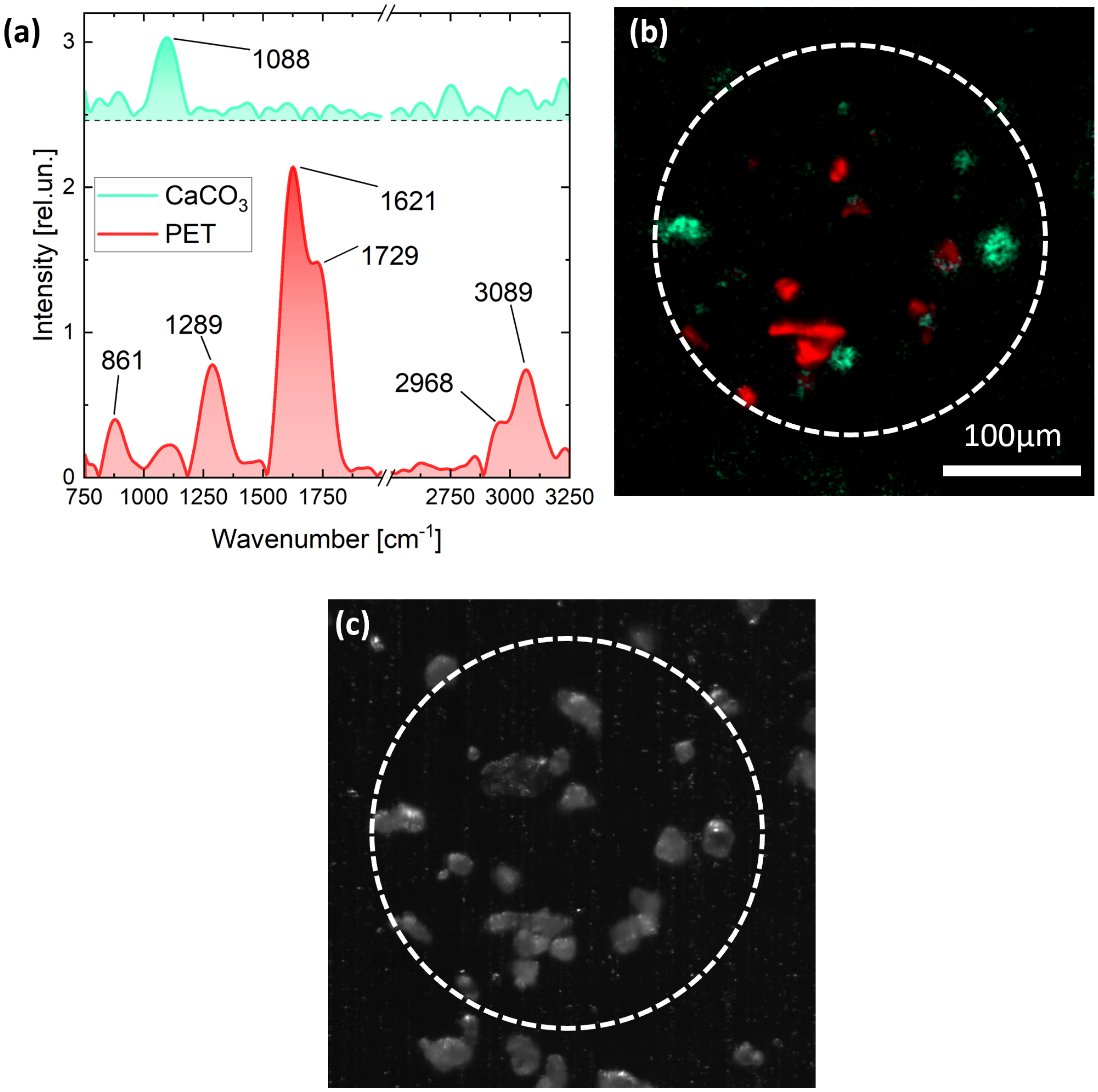

Figure S2: Wide-field low-resolution Raman microscopy of environmental PET-bottle MPs.(a) Spectra selected in regions of interest on $CaCO_3$ and PET respectively. (b) Map obtained from N-FINDR analysis on spectral hypercube. The white dashed circumference identifies the illuminated area of the sample. Number of pixels: 251.5 kpixels. Irradiance on the sample: 270 W/cm^2. Total measurement time: 7 min 58s. (c) Reflectivity image of the same FOV of panel (b) acquired with visible illumination. The white dashed circumference identifies the laser spot location during the Raman measurements.

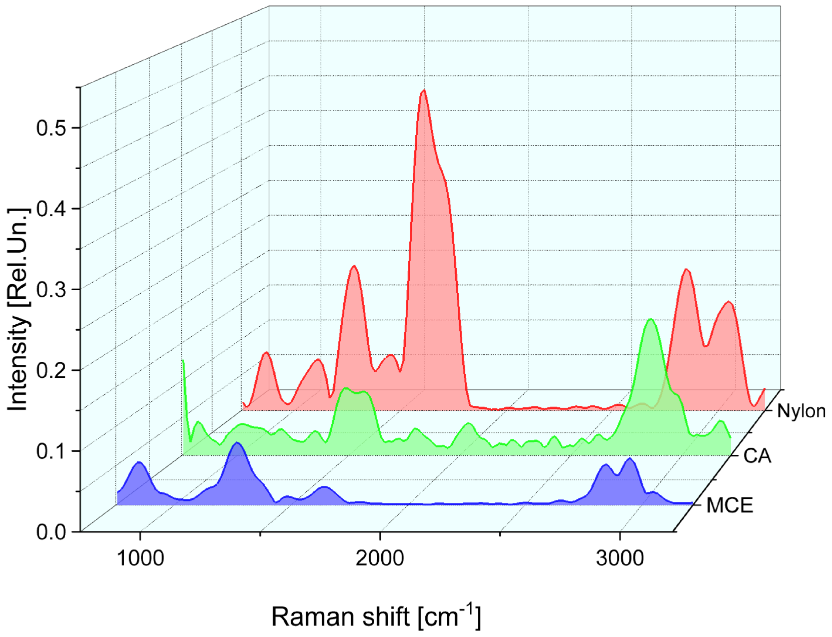

Figure S3: Spectra from each filter measurement obtained by averaging pixels in the corre-sponding FT-HSM images. Spectra have been normalized on CCD binning and integration time.

Low-resolution Raman of mixed environmental MPs

As in the case of PET-bottle MPs sample, we performed a low-resolution Raman measurement of mixed environmental MPs (high-resolution measurement in Figure 4 in the main text). Even in this case this preliminary acquisition allows to distinguish the MPs in the FOV (see Figure S4).

Comparison of MPs with other species

To corroborate the advantage of rapid low-resolution FT-HSM acquisition we compared the commercial MPs spectra shown in Figure 2 of main text with the Raman spectra of $CaCO_3$ and MCE filter measured in the same conditions. On the base of their detected Raman bands (Figure S5), MPs polymers are clearly distinguishable from these other molecules even in this low spectral resolution case.

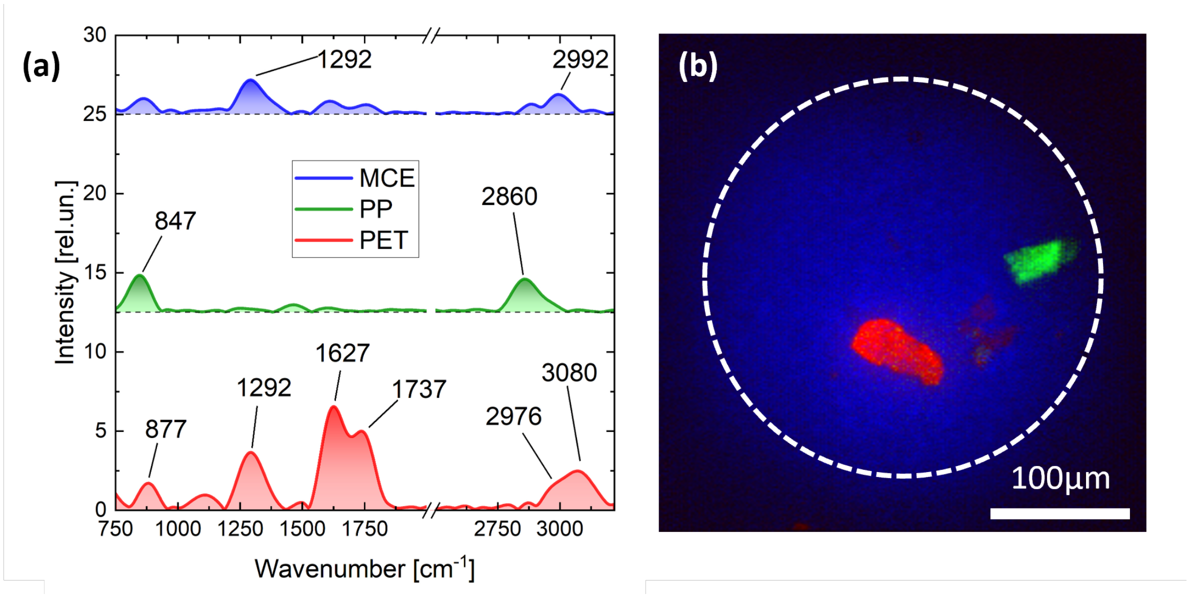

Figure S4: Wide-field low-resolution Raman microscopy of a mix of environmental MPs on MCE filter. (a) Endmember spectra retrieved by N-FINDR analysis. (b) Composite map of the endmember spectra shown in panel (a). The white dashed circumference identifies the illuminated area of the sample. Number of pixels: 62.5 kpixels. Irradiance on the sample: 270 W/cm^2. Total measurement time: 4 min 12s.

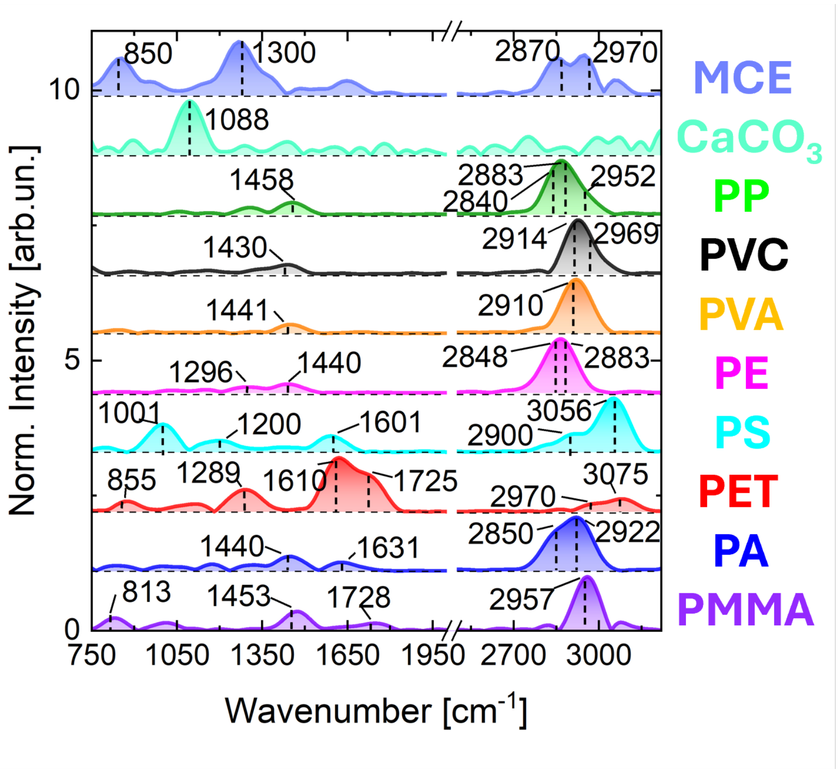

Figure S5: Spectra shown in Figure 2 of main text compared with the measured Raman signature of MCE and $CaCO_3$.

Table S1: Acquisition parameters for the filters measurements.

	MCE	CA	NYLON
CCD Binning	2×2	1×1	2×2
Number of pixels (×1000)	251.5	1000	251.5
Laser power on sample [mW]	~ 450	~ 450	~ 450
Integration time [ms]	1000	450	500
Total acquisition time [min]	20	11	32

Fluorescence measurement on PET-bottle MPs sample

We exploited the multimodal capabilities of the FT-HSM, as described in Ardini et al.,[1] to retrieve a fluorescence map of the same FOV of Raman measurement on PET-bottle MPs (Figure 3 in the main text). The measurement parameters are listed in Table S2. For this measurement we exploited the setup shown in Figure 1 of the main text without the 650-nm SPF: this little modification in the detection branch allowed us to acquire a broader spectral band with respect to the one where the Raman peaks are located. As shown in Figure S6, the fluorescence spectra of the measured particles show differences even in areas where the Raman measurement (Figure 3 in the main text) shows the same signature. These differences could be attributed to contaminants absorbed by the particles or to dyes applied to the MPs.

Table S2: Acquisition parameters for the fluorescence measurement on PET-bottle MPs sam-ple compared with the ones of high-resolution Raman measurement. Scan range and step are calculated considering 600-nm wavelength as reference.

	Fluorescence	Raman
CCD Binning	2×2	2×2
Pixel resolution [μm]	~ 0.8	~ 0.8
Number of pixels (×1000)	251.5	251.5
Irradiance on sample [W/cm^2]	~ 29	~ 285
Scan range [fs]	$-163 \rightarrow +163$	$+34 \rightarrow +2524$
Step [fs]	0.813	0.813
Number of acquired frames	400	3064
Spectral resolution [nm]	6.7	0.8
Total acquisition time [min]	2	36

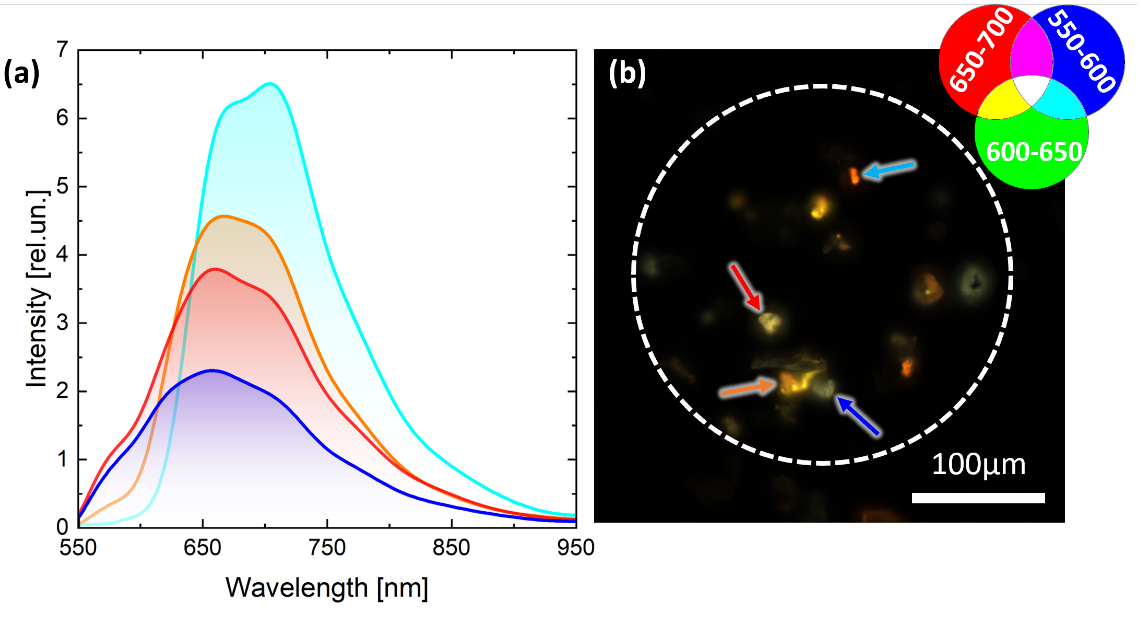

Figure S6: Wide-field fluorescence microscopy on the same FOV of Raman measurement shown in Figure 3 of the main text. (a) Spectra selected in ROIs on particles indicated by arrows in panel (b). (b) False color RGB image of acquired FOV in the range 550-700 nm (color code shown on the top right). Total measurement time: 2 min.

Raman measurement of the MCE filter obtained after vacuum filtration (blank sample)

To prevent and check possible cross-contamination or microplastics/microfibers contamination from external sources, we performed a Raman measurement of a control sample (blank sample) of filter obtained after vacuum filtration of seawater sample without spiked MPs. Figure S7 shows the result of this measurement where it is clear the absence of external contaminants and foreign particles.

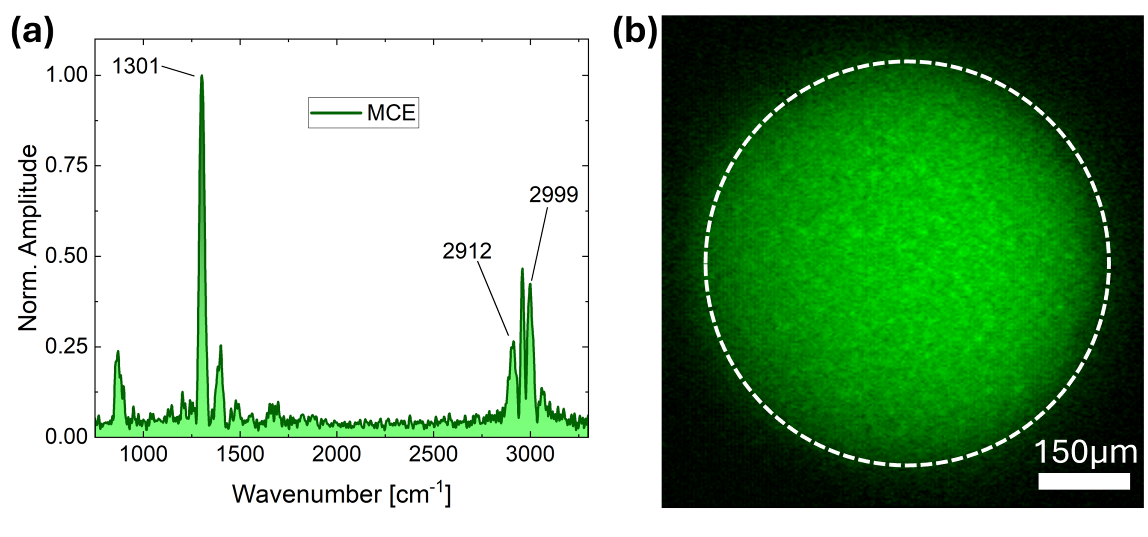

Figure S7: Wide-field Raman microscopy of MCE filter obtained after vacuum filtration. (a) Endmember spectrum retrieved by N-FINDR analysis. (b) Map of the endmember spectrum shown in panel (a). The white dashed circumference identifies the illuminated area of the sample. Objective: 10×. Number of pixels: 62.5 kpixels. Irradiance on the sample: 44 W/cm^2. Total measurement time: 31 min.

Comparison with Standard Raman spectroscopy measurements

Standard spontaneous Raman measurements on PMMA were performed using a home-built Raman microscope. The illumination light, provided by a continuous-wave diode laser centered at 660 nm (Cobolt AB, Flamenco, Solna, Sweden) is filtered using a shortpass filter (FESH0750, ThorLabs, Newton, NJ, USA), to remove possible spurious emissions, expanded by a beam expander (GBE05-B, ThorLabs, Newton, NJ, USA) and then reflected by a single-edge dichroic beamsplitter (Di03-R660-t1-25x36, Semrock, Inc., Rochester, NY, USA) aiming to separate illumination and emitted Raman photons. The light enters the back port of a commercial inverted microscope (IX73, Olympus Europa SE & Co. KG, Hamburg, Germany) and is focused on the sample using a dry/air 50x objective (MPLFLN50X 20x/0.80 NA, Olympus). The scattered light is collected by the same objective and transmitted by the dichroic beamsplitter. A long-pass (664 nm) edge filter (LP02-664RU-25, Semrock, Inc.)

is then used to remove the residual laser light and the scattered light is focused by a lens ($f = 35\,\text{mm}$, AC254-035-B-ML, ThorLabs, Newton, NJ, USA) on the entrance slit of a spectrometer (Isoplane160, Princeton instruments, Trenton, NJ, USA) equipped with a silver coated grating of 660 gr/mm and connected to a front illuminated CCD (PIXIS256F, Princeton Instruments, Trenton, NJ, USA). The wavenumber calibration was performed using toluene and a ArHg lamp (AvaLight CAL-MINI, Avantes, Apeldorn, The Netherlands) as references. Intensity calibration was performed using a calibrated white lamp (AvaLight HAL-CAL-MINI, Avantes, Apeldorn, The Netherlands). The illumination power used to collect spectra from PMMA beads was 50mW (corresponding to 0.8 MW/cm^2 irradiance) and the spectra were collected by integrating the signal for 5s, averaging 5 acquisitions. Spectral data were only calibrated and corrected for eventual cosmic rays, without performing further processing.

The same sample was measured with our FT-HSM. We used a 20× objective (Leica®PL FLUOTAR, 20×, NA 0.40), which leads to an illumination spot on the object plane with a diameter of 320 μm. The laser power was set to 350 mW, corresponding to an irradiance on the sample of 435 W/cm^2. We used a hardware 2x2 pixels binning, resulting in images with 502× 501 pixels. Since the size of the binned pixels is comparable to the Abbe's limit of the microscope, binning does not affect the spatial resolution, but increases the SNR for a given measurement time. The total measurement time, including the communication time with the motor and the camera, was 38 minutes. For the sake of comparison, in a raster-scanning approach such acquisition time would correspond to a pixel dwell time of 9 ms, resulting in unacceptably low SNR for the Raman spectra.

The comparison in Figure S8 shows a good agreement between the spectra obtained from the FT-HSM measurement. In addition, the spectrum from the standard Raman microscope has a strong fluorescence background, which is not present in the data from the HSM micro-scope. The correspondence of the characteristic Raman peaks of PMMA between the two measurements demonstrates that the approach of our HSM for the fluorescence subtraction

is not affecting the measurement of the Raman peaks.

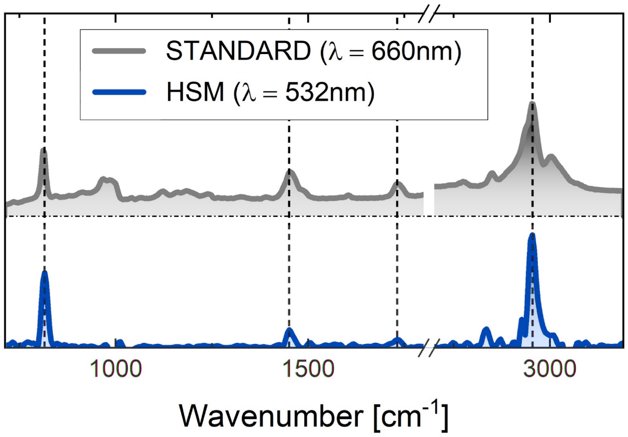

Figure S8: Comparison between spectra obtained with standard Raman spectrometer (grey) and with Raman HSM measurements (blue) for PMMA beads. The dashed lines highlight the characteristic Raman peaks of PMMA. The two spectra have been offset in vertical axis for clarity.

μATR-FTIR spectroscopy of MPs on filters

To validate the accuracy of the FT-HSM results, we also examined the filters under the stereomicroscope (magnification up to 65×) and the identified particles were analyzed with the μATR-FTIR spectroscopy (Spotlight 200i FT-IR microscope system, Perkin Elmer), widely established as effective method for MPs characterization. The measurements confirm that the Raman FT-HSM had actually detected and identified all the particles present on the filter in the same field of view. The images of two MPs fragments and their IR absorption spectra results are shown in Figure S9.

References

(1) Ardini, B.; Bassi, A.; Candeo, A.; Genco, A.; Trovatello, C.; Liu, F.; Zhu, X.; Valentini, G.; Cerullo, G.; Vanna, R.; others High-throughput multimodal wide-field Fourier-transform Raman microscope. *Optica* **2023**, *10*, 663–670.

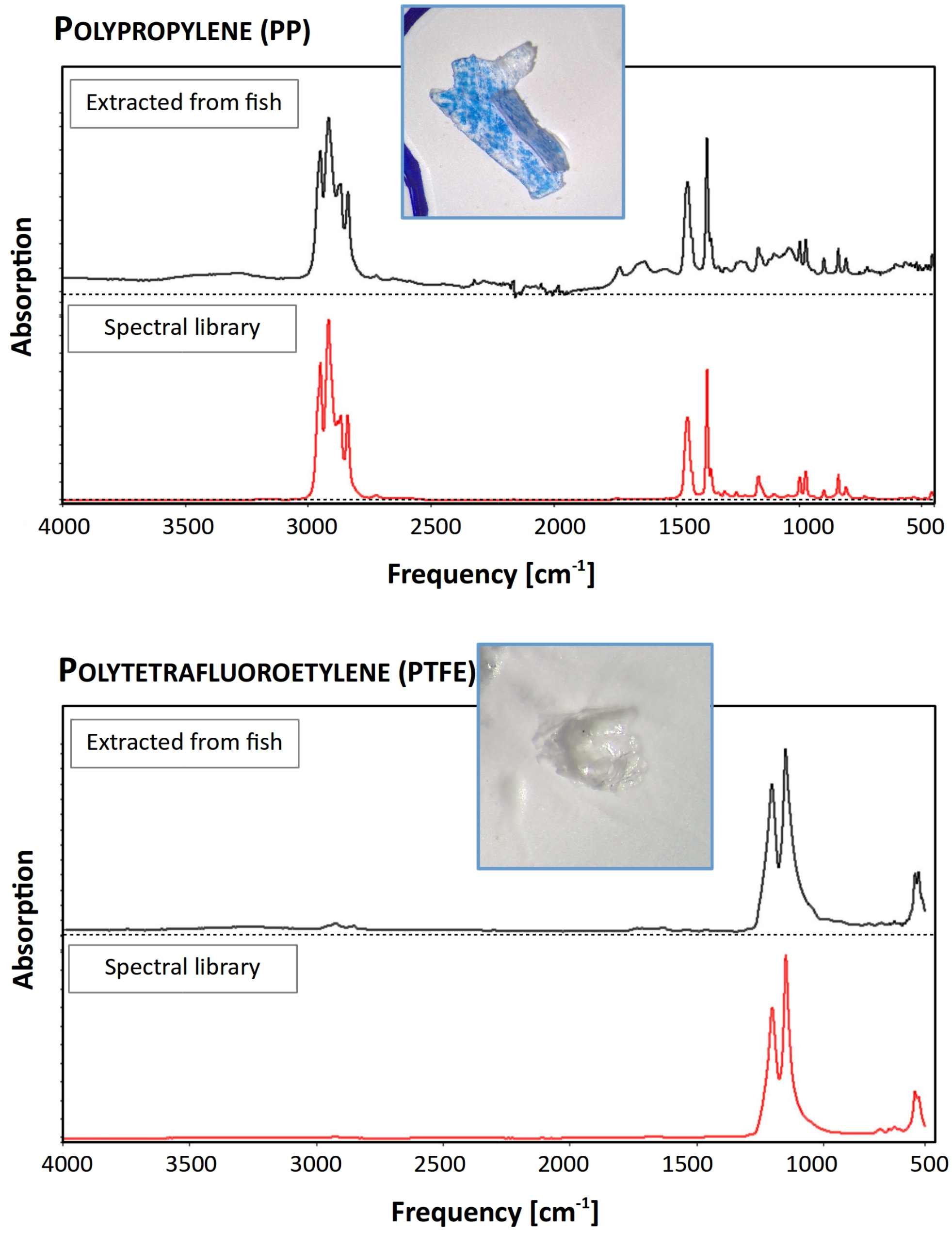

Figure S9: Images of MPs extracted from fish and the relative IR spectra (in black) acquired through μATR-FTIR microscope system. The red spectra are obtained from spectral li-braries for polymer identification.
